# Draft Genome Sequences of Enterohemorrhagic *Escherichia coli* Encoding Extended-Spectrum Beta-Lactamases

**DOI:** 10.1128/genomeA.01633-15

**Published:** 2016-02-11

**Authors:** Charlotte Valat, Robert J. Goldstone, Edouard Hirchaud, Marisa Haenni, David G. E. Smith, Jean-Yves Madec

**Affiliations:** a Anses, Laboratoire de Lyon, Unité Antibiorésistance et Virulence Bactériennes, Lyon, France; b School of Life Sciences, Heriot-Watt University, Edinburgh, United Kingdom; c Anses, Laboratoire de Ploufragan-Plouzané, Unité Génétique Virale et Biosécurité, Plouzané, France

## Abstract

Extended-spectrum beta-lactamases (ESBLs) have rarely been observed among Shiga toxigenic *Escherichia coli* (STEC), and, to our best knowledge, only three ESBL-positive isolates of the enterohemorrhagic *E. coli* (EHEC) subpathotype have been reported. Here, we present the first draft genome sequences of two ESBL-positive EHEC isolates belonging to serotypes O111:H8 and O151:H16.

## GENOME ANNOUNCEMENT

Shiga toxigenic *Escherichia coli* (STEC), including the subpathotype of enterohemorrhagic *E. coli* (EHEC) carrying *stx* and *eae* genes, is frequently associated with human infection. Ruminants are the major source of STEC, and they are also a reservoir of extended-spectrum beta-lactamase (ESBL)-producing *E. coli*, mostly of the CTX-M type (1). ESBLs have been associated with STEC in rare cases, either in humans (O104:H4/CTX-M-15 [2], O26/TEM-52 [3], O64/CTX-M-3 [4], O157:H7/CTX-M-1 [5], and O26:H11/CTX-M-18 [6]), in chickens (O157/CTX-M-2 [7]), or in cattle (O111:H8/CTX-M-15 [8] and O145:NM/CTX-M-1 [9]); however, to the best of our knowledge, only 3 ESBL-producing *E. coli* strains belonging to the subpathotype of EHEC (STx and Eae) have been identified so far (6, 8, 9). The aim of this study was to present the first draft genome sequences of two ESBL-producing EHEC, including those belonging to the serotype O111:H8 (sequence type 16 [ST16]) first characterized using a DNA array and PCR in a previous study (8), and a new isolate of serotype O151:H16 (ST21) producing CTX-M-1 and carrying the *stx1a* and *eaeβ1* genes. These two ESBL-producing EHEC isolates were isolated from diseased cattle in France through the national surveillance network of antimicrobial resistance in animal pathogens (Resapath [http://www.resapath.anses.fr]).

Genomic DNA was extracted from overnight culture using the MasterPure DNA purification kit (Epicentre). Sequencing was performed using an Illumina MiSeq sequencer at the Wolfson Wohl Cancer Research Centre, United Kingdom. A multiplex sequencing approach was used, involving 12 separately tagged libraries sequenced simultaneously in two lanes. The standard Illumina indexing protocol involved the fragmentation of 2 µg of genomic DNA by acoustic shearing to enrich for 200-bp fragments. A-tailing, adapter ligation, and an overlap extension PCR using the Illumina 3-primer set were performed to introduce specific tag sequences between the sequencing and flow cell binding sites of the Illumina adapter. DNA cleanup was carried out after each step to remove DNA sequences <150 bp using AMPure paramagnetic beads (Beckman Coulter, Inc., USA), and a quantitative PCR (qPCR) was used for final DNA quantification. The raw reads were trimmed by the removal of ambiguous nucleotides from the read ends and reads for which quality scores were <0.001. Reads <15 nucleotides were also removed. For *de novo* assembly using CLC Genomics Workbench (version 6.5.2), scaffolding was performed, and paired distances were automatically detected. The minimum contig length was set to 200 bp. The *de novo* assembly produced 822 contigs for *E. coli* O111:H8 (isolate #22207) and 818 contigs for *E. coli* O151:H16 (isolate #22593). The median coverages of the assemblies were 110× for #22207 and 106× for #22593, with an *N*_50_ of 97 kbp and 85 kbp, and a genome size of 5.607 and 5.868 Mb, respectively.

The contigs were annotated using the National Center for Biotechnology Information (NCBI) Prokaryotic Genome Annotation Pipeline (PGAP) at http://www.ncbi.nlm.nih.gov/genomes/static/Pipeline.html. In the genomes of #22207 and #22593, 5,567 and 5,837 coding sequences were identified, respectively. Future comparative analysis of whole-genome sequencing data from these isolates will provide insights into the attributes of emerging ESBL-producing EHEC.

### Nucleotide sequence accession numbers.

The whole-genome shotgun projects have been deposited at DDBJ/EMBL/GenBank under the accession numbers LMBJ00000000 and LMBK00000000. The versions described in this paper are versions LMBJ01000000 and LMBK01000000, respectively.

## References

[1] Valat C, Auvray F, Forest K, Métayer V, Gay E, Peytavin de Garam C, Madec JY, Haenni M 2012 Phylogenetic grouping and virulence potential of extended-spectrum-beta-lactamase-producing *Escherichia coli* strains in cattle. Appl Environ Microbiol 78:4677–4682. doi:10.1128/AEM.00351-12. 22522692 PMC3370483

[2] Mellmann A, Harmsen D, Cummings CA, Zentz EB, Leopold SR, Rico A, Prior K, Szczepanowski R, Ji Y, Zhang W, McLaughlin SF, Henkhaus JK, Leopold B, Bielaszewska M, Prager R, Brzoska PM, Moore RL, Guenther S, Rothberg JM, Karch H 2011 Prospective genomic characterization of the German enterohemorrhagic *Escherichia coli* O104:H4 outbreak by rapid next generation sequencing technology. PLoS One 6:e22751. doi:10.1371/journal.pone.0022751. 21799941 PMC3140518

[3] Buvens G, Bogaerts P, Glupczynski Y, Lauwers S, Piérard D 2010 Antimicrobial resistance testing of verocytotoxin-producing *Escherichia coli* and first description of TEM-52 extended-spectrum β-lactamase in serogroup O26. Antimicrob Agents Chemother 54:4907–4909. doi:10.1128/AAC.00551-10. 20733038 PMC2976167

[4] Dutta TK, Warjri I, Roychoudhury P, Lalzampuia H, Samanta I, Joardar SN, Bandyopadhyay S, Chandra R 2013 Extended-spectrum-beta-lactamase-producing *Escherichia coli* isolate possessing the Shiga toxin gene (*stx*_1_) belonging to the O64 serogroup associated with human disease in India. J Clin Microbiol 51:2008–2009. doi:10.1128/JCM.00575-13. 23576543 PMC3716047

[5] Torpdahl M, Nielsen EM, Scheutz F, Olesen B, Hansen DS, Hasman H 2013 Detection of a Shiga toxin- and extended-spectrum-beta-lactamase-producing *Escherichia coli* O157:H7 human clinical isolate. J Antimicrob Chemother 68:1203–1204. doi:10.1093/jac/dks516. 23325753

[6] Ishii Y, Kimura S, Alba J, Shiroto K, Otsuka M, Hashizume N, Tamura K, Yamaguchi K 2005 Extended-spectrum beta-lactamase-producing Shiga toxin gene (Stx1)-positive *Escherichia coli* O26:H11: a new concern. J Clin Microbiol 43:1072–1075. doi:10.1128/JCM.43.3.1072-1075.2005. 15750063 PMC1081271

[7] Roest HI, Liebana E, Wannet W, van Duynhoven Y, Veldman KT, Mevius DJ 2007 Antibiotic resistance in *Escherichia coli* O157 isolated between 1998 and 2003 in The Netherlands. Tijdschr Diergeneeskd 132:954–958. 18225714

[8] Valat C, Haenni M, Saras E, Auvray F, Forest K, Oswald E, Madec JY 2012 CTX-M-15 extended-spectrum beta-lactamase in a Shiga toxin-producing *Escherichia coli* isolate of serotype O111:H8. Appl Environ Microbiol 78:1308–1309. doi:10.1128/AEM.06997-11. 22156432 PMC3273006

[9] Ewers C, Stamm I, Stolle I, Guenther S, Kopp PA, Fruth A, Wieler LH, Scheufen S, Bauerfeind R, Bethe A, Prenger-Berninghoff E 2014 Detection of Shiga toxin- and extended-spectrum beta-lactamase-producing *Escherichia coli* O145:NM and Ont:NM from calves with diarrhoea. J Antimicrob Chemother 69:2005–2007. 24595804 10.1093/jac/dku042

